# Young Bone Marrow Sca-1 Cells Rejuvenate the Aged Heart by Promoting Epithelial-to-Mesenchymal Transition: Erratum

**DOI:** 10.7150/thno.81626

**Published:** 2023-01-09

**Authors:** Jiao Li, Shu-Hong Li, Jun Wu, Richard D. Weisel, Alina Yao, William L. Stanford, Shi-Ming Liu, Ren-Ke Li

**Affiliations:** 1 Department of Cardiology, Second Affiliated Hospital of Guangzhou Medical University, Guangzhou, China; 2 Toronto General Research Institute, Division of Cardiovascular Surgery, University Health Network, Toronto, Canada; 3 Division of Cardiac Surgery, Department of Surgery, University of Toronto; Toronto, Canada; 4 Regenerative Medicine Program, Ottawa Hospital Research Institute, Department of Cellular and Molecular Medicine, University of Ottawa

The authors regret that the original version of our paper, unfortunately, contained incorrect pictures in Figure 3E, for the 3^rd^ image of the transwell migration (E+Sca1^+^) assay, as well as in Figure 5F for the 1^st^ (E), 3^rd^ (E+Sca1^+^) and 4^th^ (E+TGF-β1) images of the transwell migration assay. The corrected images for Figure 3E and Figure 5F are provided below.

The correction made in this erratum does not affect the original data and conclusions. The authors apologize for any inconvenience that the errors may have caused.

## Figures and Tables

**Figure 3:**
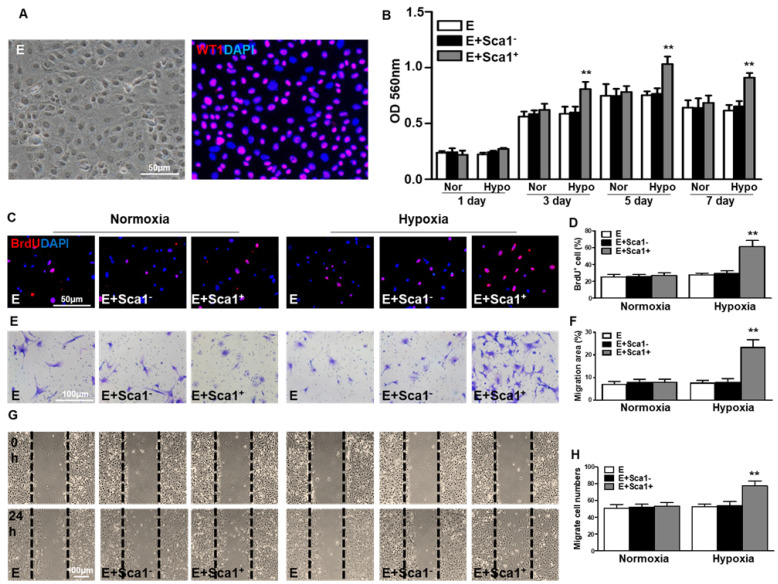
** Co-culture of BM Sca-1^+^ cells with EPDCs under hypoxia conditions increased proliferation and migration of EPDCs.** Epicardial-derived cells (EPDCs, abbreviated as E) were isolated and co-cultured with bone marrow (BM) Sca-1^+^ cells or Sca-1^-^ cells under normoxia and hypoxia (0.1% O_2_) conditions. **(A)** Representative images of EPDCs and immunofluorescent labeling of WT1. **(B)** Cell proliferation was determined by the MTT (3-(4,5-dimethylthiazol-2-yl)-2,5-diphenyltetrazolium bromide) assay. **(C, D)** Immunofluorescent staining of BrdU and quantification of BrdU^+^ (proliferating epicardial cells) cells. EPDCs, co-cultured with BM Sca-1^+^ cells or Sca-1^-^ cells under normoxia (Nor) and hypoxia (Hypo) conditions for 72 h, were pulse-chased with BrdU (10 µM) for labeling of proliferative cells. Cell migration was evaluated by the transwell **(E, F)** and wound-scratch **(G, H)** assays after co-culture for 24 h. n=6/group, mean ± SD; **P<0.01. BrdU: bromodeoxyuridine.

**Figure 5:**
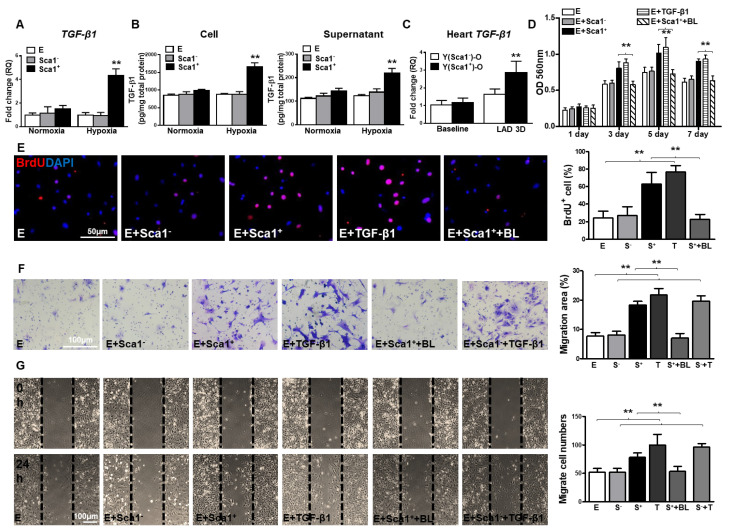
** BM Sca-1^+^ cells increased proliferation and migration of EPDCs through TGF-β1 signaling.** Epicardial-derived cells (EPDCs, abbreviated as E), bone marrow (BM) Sca-1^+^ and Sca-1^-^ cells were isolated and subjected to normoxia and hypoxia (0.1% O_2_) conditions. **(A)** Higher expression levels of TGF-β1 mRNA were found in BM Sca-1^+^ cells than in EPDCs and Sca-1^-^ cells under hypoxia conditions for 72 h. **(B)** TGF-β1 homodimer secretion in EPDCs, BM Sca-1^+^ and Sca-1^-^ cell lysate and culture medium under normoxia and hypoxia conditions for 72 h was quantified by ELISA. **(C)** TGF-β1 mRNA expression was measured in the Y(sca-1^+^)-O and Y(sca-1^-^)-O chimeric hearts at baseline and in the infarcted area at 3 days post-MI. Epicardial-derived cells (EPDCs, abbreviated as E) were co-cultured with BM Sca-1^-^ cells (S^-^), Sca-1^+^ cells (S^+^), TGF-β1 (T, 5 ng/mL), Sca-1^+^ cells with TGF-β1 blocking antibody (S^+^+BL, 1 µg/mL) or Sca-1^-^ cells with TGF-β1 (S^-^+T) under hypoxia conditions. The following assays were conducted in EPDCs: **(D)** MTT assay measured cell proliferation; **(E)** EPDCs, co-cultured with BM Sca-1^+^ cells or Sca-1^-^ cells under normoxia and hypoxia conditions for 72 h, were pulse-chased with BrdU (10 µM) for labeling of proliferative cells; **(F)** transwell and **(G)** wound-scratch assays measured migration areas and number of EPDCs after co-culture for 24 h, respectively. Insufficiency of Sca1^-^ cells on EPDC migration can be rescued by TGF-β1, and EPDC migration was restored to a level comparable with that of the Sca-1^+^ cell- or TGF-β1-treated groups. n=6/group, mean ± SD; **P<0.01. BrdU: bromodeoxyuridine; MTT: 3-(4,5-dimethylthiazol-2-yl)-2,5-diphenyltetrazolium bromide.

